# Fast identification and quantification of c-Fos protein using you-only-look-once-v5

**DOI:** 10.3389/fpsyt.2022.1011296

**Published:** 2022-09-23

**Authors:** Na Pang, Zihao Liu, Zhengrong Lin, Xiaoyan Chen, Xiufang Liu, Min Pan, Keke Shi, Yang Xiao, Lisheng Xu

**Affiliations:** ^1^The College of Medicine and Biological Information Engineering, Northeastern University, Shenyang, China; ^2^Institute of Biomedical and Health Engineering, Shenzhen Institutes of Advanced Technology, Chinese Academy of Sciences, Shenzhen, China; ^3^Shenzhen Hospital of Guangzhou University of Chinese Medicine, Shenzhen, China; ^4^National Innovation Center for Advanced Medical Devices, Shenzhen, China; ^5^Key Laboratory of Medical Image Computing, Ministry of Education, Shenyang, China

**Keywords:** neuroscience, neuron activity, c-Fos, deep learning, quantitative statistics

## Abstract

In neuroscience, protein activity characterizes neuronal excitability in response to a diverse array of external stimuli and represents the cell state throughout the development of brain diseases. Importantly, it is necessary to characterize the proteins involved in disease progression, nuclear function determination, stimulation method effect, and other aspects. Therefore, the quantification of protein activity is indispensable in neuroscience. Currently, ImageJ software and manual counting are two of the most commonly used methods to quantify proteins. To improve the efficiency of quantitative protein statistics, the you-only-look-once-v5 (YOLOv5) model was proposed. In this study, c-Fos immunofluorescence images data set as an example to verify the efficacy of the system using protein quantitative statistics. The results indicate that YOLOv5 was less time-consuming or obtained higher accuracy than other methods (time: ImageJ software: 80.12 ± 1.67 s, manual counting: 3.41 ± 0.25 s, YOLOv5: 0.0251 ± 0.0003 s, *p* < 0.0001, *n* = 83; simple linear regression equation: ImageJ software: *Y* = 1.013 × *X* + 0.776, *R*^2^ = 0.837; manual counting: *Y* = 1.0*X + 0, *R*^2^ = 1; YOLOv5: *Y* = 0.9730*X + 0.3821, *R*^2^ = 0.933, *n* = 130). The findings suggest that the YOLOv5 algorithm provides feasible methods for quantitative statistical analysis of proteins and has good potential for application in detecting target proteins in neuroscience.

## Introduction

Proteins, as biological macromolecules, play an important role in life activities and are an indispensable part of scientific research, revealing the mysteries of life (1). They are involved in the regulation of gene expression (2), redox (3), neurotransmission (4), learning and memory (5), as well as other cellular activities. In neuroscience, protein research is essential. c-Fos is a type of protein that reflects proto-oncogenes; it has been extensively used as a marker for the activation of neurons (6), especially in physical neuromodulation methods of optogenetics (7), deep brain stimulation (DBS) (8), transcranial magnetic stimulation (TMS) (9), and ultrasound stimulation (10). In biology, the state of proteins can be used to evaluate cell activity (11), protein misfolding and aggregation, which can be detected to assess the development of brain diseases such as Parkinson’s disease (alpha-synuclein), Alzheimer’s disease (AD) (amyloid beta) and Huntington’s disease (huntingtin) (12). Therefore, it is important to develop a method for identifying and quantifying proteins.

Currently, there are a few studies on the quantitative statistical methods of proteins in immunofluorescence images. Manual counting and ImageJ software (13) are most commonly used, but they are laborious, time-consuming and cumbersome. Fortunately, the advent of artificial intelligence (AI) technology has overcome this dilemma. Recently, AI has been widely used in medical image analysis and has achieved state-of-the-art performance for several clinical tasks (14). For instance, Pohlen et al. (15) and Lei et al. (16) segmented multi-site infant brains based on magnetic resonance imaging (MRI) to better understand early brain development in healthy people and patients with disorders. Gao et al. (17), He et al. (18) and Zhang et al. (19) utilized deep learning methods to distinguish COVID-19 from other types of pneumonia using computed tomography (CT) or X-ray images, responding to the urgent need to treat COVID-19 patients effectively. Deep learning has been successfully exploited for object detection (20), classification (21), and synthesis of medical images (22) with remarkable results. In addition to the aforementioned success in the field of medical imaging, AI has also been applied extensively to neuroscience (23). For example, automated prediction of brain activity, such as epileptic seizures (24), dementia with Lewy bodies (DLB), AD diagnosis (25), and brain response that reveals a cortical processing hierarchy (26), are some of the applications.

You-only-look-once (YOLO) is a typical one-stage object detection algorithm (27). YOLO is characterized by high detection speed, low background error detection rate, and strong versatility (28). Moreover, after continuous optimization, YOLO has now been updated to YOLOv5, and YOLOv5 outperforms previous versions in terms of accuracy (29). YOLOv5 is the fastest and lightest among the YOLO series, and has been applied in various fields. For example, in industrial and agricultural fields, Song et al. (30) proposed a strategy to improve the positioning accuracy of grasping robots, and Zhao et al. (31) detected particleboard surface defects. Fan et al. (32) used YOLOv5 to recognize strawberry maturity. In particular, Wan et al. (33) and Mushtaq et al. (34) demonstrated the potential ability of YOLOv5 to detect lumbar spine deformities and polyps from colorectal images.

In this study, our main purpose is to apply YOLOv5 to fast identify and quantify the protein. Firstly, we constructed a model to enhance image resolution at low magnification. Subsequently, an object detection model was utilized for cell recognition. Finally, we compared the performance of the proposed YOLOv5 with two traditional methods.

## Materials and methods

### Image acquisition

Immunofluorescence images were collected from the Shenzhen Institutes of Advanced Technology, Chinese Academy of Sciences and Shanghai Key Laboratory of Psychotic Disorders. C-Fos is a marker of neuronal activation which is widely used to locate external stimulation in rodent animals (6, 35), and c-Fos images obtained by ultrasound stimulation were selected as the training data. Mice were stimulated by ultrasound for 30 min and placed in a quiet room for 60–90 min. Then, the mice were sacrificed and brain tissues were obtained. The brain tissues were cut into slices of a 20 mm thickness and pre-rinsed three times with phosphate buffer saline. The slices were permeabilized and blocked at room temperature for 1 h and incubated with primary antibodies (c-Fos, Synaptic Systems, 226003) at 4^°^C for over 12 h. The slices were then washed thrice and incubated with secondary antibodies (488 donkey anti-rabbit, ThermoFisher, Waltham, MA, USA, A21206) for 3 h. After washing, the slices were counterstained by 4’, 6-diamidino-2-phenylindole (DAPI). Images were acquired using a Nikon confocal microscope (ECLIPSE Ti2-U, Nikon, Japan).

### Data pre-processing

The pixel size of images was mostly 1,636 × 1,088 and 1,024 × 1,024, in a TIFF format. Further, the color of the images was uniformly adjusted so that green represented c-Fos and blue represented the nucleus. In addition, the contrast and saturation of fluorescence images with a fuzzy background were enhanced in terms of intensity. The images were annotated using Labelme software and proofread by two experienced technicians with more than five years of experience. The annotation box was tangential to the edge of the target cell. Some of the images needed to be enlarged because of the different magnifications. To facilitate model training and recognition we uniformly enlarged the images with small magnification to twice their original size, and the images were cut into a pixel size of 512 × 512 using the window method. Meanwhile, the super-resolution generative adversarial network (SRGAN) reconstruction (36, 37) was used to offset blurred details after image enlargement. The images with a size of 512 × 512 formed a dataset conducive to the parameter calculation of the model. The dataset was randomly divided into a training set (80%) and a test set (20%).

### Network system

#### Super-resolution reconstruction

Generative adversarial networks (GANs) consist of two models: a generator and a discriminator that act against each other to produce good outputs. The generator network purports to create samples that are as realistic as possible. The discriminator network then determines whether the image is from a real or fake sample set. Super-resolution reconstruction was a difficult task until Ledig et al. (38) proposed a GAN-related network in this field. SRGAN utilizes perceptual losses, including adversarial and content losses, and uses a discriminative network to distinguish the reconstructed image from the original one. The output image reaches the level of photorealism.

#### Model training

As a representative approach for object detection, the YOLOv5 model was used for training, and the specific model used was YOLOv5l which is ideal for detecting small objects (39). The object detection task was treated as a regression problem, and the coordinates of the bounding box, confidence degree, and category probability of objects contained in the box were obtained directly from the pixels of the entire image. YOLOv5 can quickly complete object detection tasks. The parameters were fine-tuned by our datasets, and the batch, image size, epoch, and learning rate were 64, 512 × 512, 100, and 0.001, respectively. Without a complex detection process, the detection results could only be obtained by inputting images into the neural network. To evaluate this model, we utilized the recall and precision formulas, which are defined as follows:


(1) $$\begin{array}{c} \text{Precision}=\frac{\text{TP}}{\text{TP}+\text{FP}} \end{array}$$

(2) $$\begin{array}{c} \text{Recall}=\frac{\text{TP}}{\text{TP}+\text{FN}} \end{array}$$

where TP is a positive sample predicted to be a positive sample, FN is a negative sample predicted to be a negative sample, FP is a negative sample predicted to be a positive sample, and TN is a positive sample predicted to be a negative sample.

**FIGURE 1:**
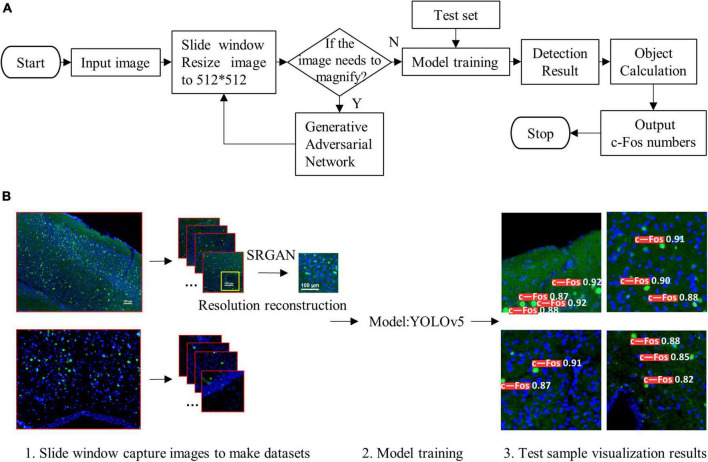
Pipeline of the proposed algorithm. **(A)** Simple flow chart. **(B)** Flow chart of visualization. The proposed system (1) used sliding window and resized the input images to 512 × 512, (2) reconstructed resolution of images, (3) trained detection model, and (4) outputted visualization results.

**FIGURE 2:**
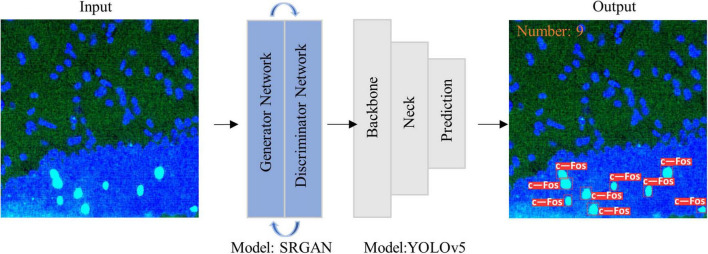
Diagram of model structure. Images were imported into super-resolution generative adversarial network (SRGAN) model, and then the enlarged image was inputted to you-only-look-once-v5 (YOLOv5) model to obtain visualization results as output.

In addition, the average precision (AP) is the area under the precision-recall curve and calculated as follows:


(3) $$\begin{array}{c} \frac{1}{n}\,\sum m\,a\,x\,{\left(p\,\left(r\,\left(k\right)\right)\right)}^{*}\,\left(r\,\left(k\right)-r\,\left(k-1\right)\right),r\in \left\{0,r\left(0\right),r\left(1\right),.r\left(k\right),1\right\} \end{array}$$

where *r* (*k*) is the *k*th recall rate, and *p* (*r* (*k*)) is the precision rate of *r* (*k*). The pipeline of the proposed algorithm and diagram of model structure were shown in Figures 1A, B, 2, respectively.

#### Data post-processing

After model training, the original image was input for identifying. The area of interest was manually calibrated or the entire graph was counted according to the actual situation. When the entire graph was input, the image was windowed and input into the network model. When the input end was the manual calibration area, the minimum enclosing rectangle was considered for the polygon area, and the pixel points of the non-interesting area were set to 0. Finally, the detection result was saved according to its confidence value, and calculated the number of boxes as the final counting. After testing processing, the reliable result including processing time, and numbers, saved as a readable file for statistical analysis.

#### ImageJ software processing

To evaluate the performance of YOLOv5, ImageJ was used as a comparison. The specific processing steps are as follows: (1) the image was imported into ImageJ software, converted from RGB color into composite, and split into three channels; (2) the c-Fos and DAPI channels were automatically segmented using “Minimum Threshold” and “Mean Threshold,” and created selections, respectively; (3) selections were added in the region of interest (ROI) manager and overlap was obtained by merged ROIs; (4) the overlapping selection was converted into a binary image by creating mask; (5) the binary image was analyzed by combining the “Analyze Particles” function with a flexible preset size range and circularity to remove false positives and obtain the protein count.

#### Statistical analysis

All data from the same group are presented as mean ± SD values. An independent samples *t*-test was performed to compare the results of the different methods. The statistical significance was set at *p* < 0.05.

## Results

### Super-resolution reconstruction of images by super-resolution generative adversarial network

**FIGURE 3:**
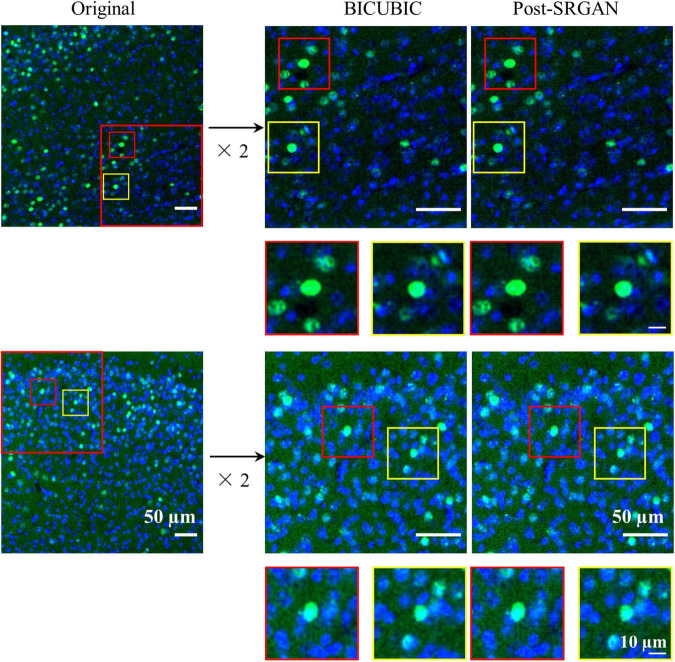
The images before and after super-resolution generative adversarial network (SRGAN). Left panels represent the original images. Middle panels are the representative BICUBIC images. Right panels show the images of post-SRGAN.

Owing to the different magnifications of the images, we enlarged them to facilitate model training and recognition. However, the magnified images showed blurred edges, which were not beneficial for identifying the target proteins. Therefore, we utilized the SRGAN model to reconstruct the images, and then the model was trained. The pipeline and model structure were shown in Figures 1, 2, respectively. And the results showed that the post-SRGAN images obtained more details and higher spatial resolution (Figure 3).

### Precision-recall curve of target protein

**FIGURE 4:**
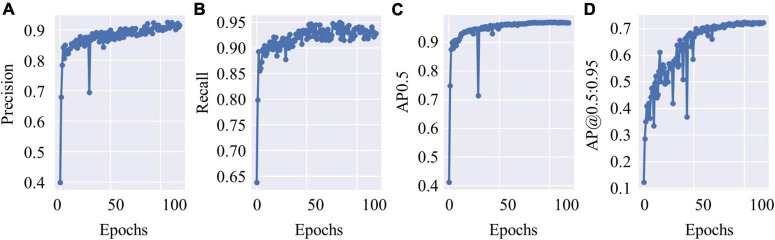
The test results of you-only-look-once-v5 (YOLOv5). **(A)** The precision values of YOLOv5 during test with epochs. **(B)** The recall values of YOLOv5 during test with epochs. **(C)** Average precisions (AP) values with the intersection over union (IoU) threshold value as 0.5. **(D)** AP values with different IoU threshold values that range from 0.5 to 0.95 with 0.05 step size.

**FIGURE 5:**
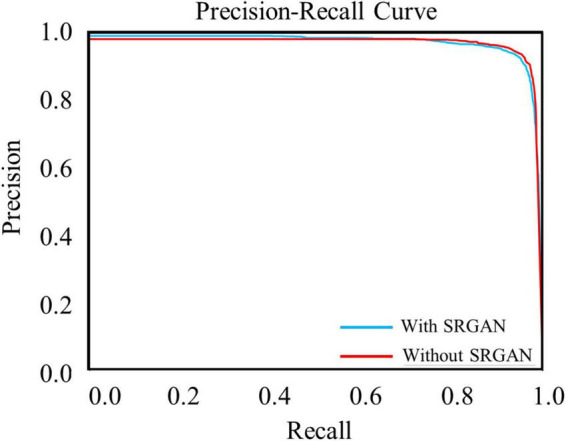
Precision-recall curves of target protein with (blue line) and without (red line) super-resolution generative adversarial network (SRGAN) for intersection over union (IoU) threshold of 0.5.

The test data constituted approximately 20% of the dataset, which contained 4,530 c-Fos images with a size of 512 × 512. During the test, the precision values and recall values were calculated with the epochs that range of 0–100, and the AP was calculated at different Intersection over Union (IoU) thresholds, as shown in Figures 4A–D. AP0.5 represents AP values for an IoU threshold of 0.5, and AP@0.5:0.95 represents AP values with different IoU threshold from 0.5 to 0.95 with 0.05 step size. The precision-recall curve for an IoU threshold of 0.5 is presented in Figure 5. As shown in Table 1, APs were assigned for different IoU thresholds with or without SRGAN. When the IoU threshold was set to 0.75, the APs had the greatest contrast between SGRAN and BICUBIC.

**TABLE 1:** Average precisions (APs) for different intersection over union (IoU) thresholds based on super-resolution generative adversarial network (SRGAN) and BICUBIC.

Image processing	AP@0.5:0.05:0.95	AP0.5	AP0.75
SRGAN	72.4	97.1	95.6
BICUBIC	61.3	96.1	76.9

### Results of different recognition methods

**FIGURE 6:**
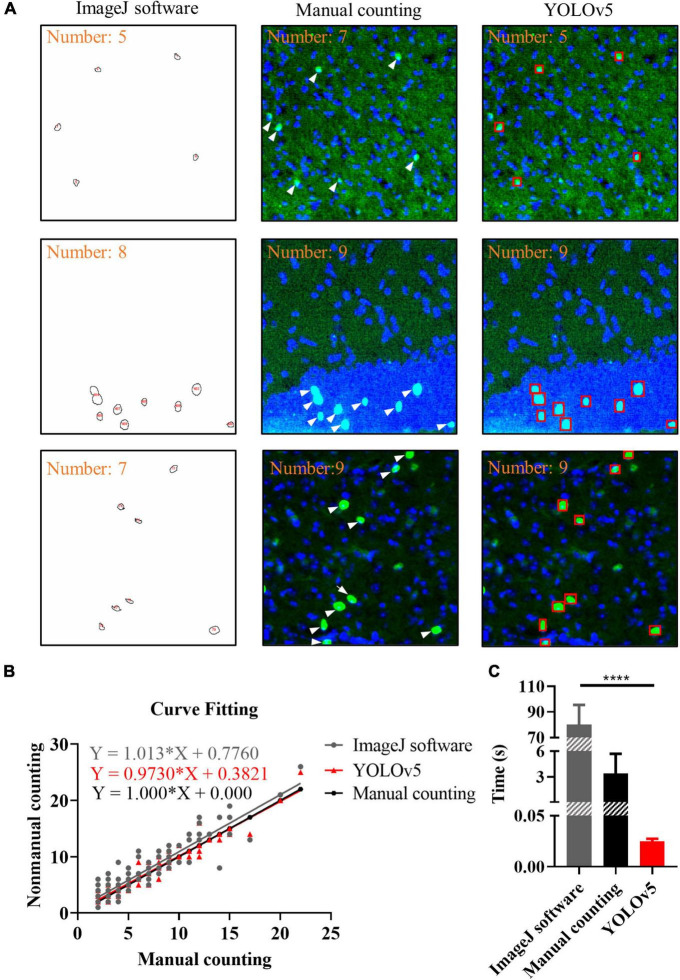
Results of different recognition methods. **(A)** Representative images of c-Fos recognition processed by ImageJ software (closed circles), manual counting (white arrows) and you-only-look-once-v5 (YOLOv5) (red boxes). **(B)** Curve fitting between different methods. The dots show the data and lines represent fitting curves. **(C)** Processing time for different methods. The data shown represent the mean ± SD values for the indicated *n*. ****p < 0.0001, from an independent samples *t*-test.

To evaluate the performance of YOLOv5, the ImageJ and manual counting were used for comparison. We used manual counting, ImageJ, and YOLOv5 to process the same batch of images. For ImageJ, we used Threshold algorithm and Watershed algorithm, the results showed that there was no significant difference between the two algorithms (Supplementary Figures 1, 2). As shown in Figure 6A, there were representative images of c-Fos recognition processed by manual counting, ImageJ software and YOLOv5; the results showed that more positive c-Fos cells were detected by AI when YOLOv5 was compared with ImageJ software. Curve fitting of AI showed that most data points were evenly distributed on either side of the fitting curve whereas some data points were located far away from the fitting curve in the ImageJ method. The fitting curves indicated that YOLOv5 was more accurate than the ImageJ software (simple linear regression equation: ImageJ software: *Y* = 1.013 × *X* + 0.776, *R*^2^ = 0.837; manual counting: *Y* = 1.0*X + 0, *R*^2^ = 1; YOLOv5: *Y* = 0.9730*X + 0.3821, *R*^2^ = 0.933, *n* = 130; Figure 6B). Meanwhile, bar graphs indicated that YOLOv5 took the shortest time in protein target identification (time: ImageJ software: 80.12 ± 1.67 s, manual counting: 3.41 ± 0.25 s, YOLOv5: 0.0251 ± 0.0003 s, *p* < 0.0001, *n* = 83, Figure 6C).

## Discussion

In this study, we used the YOLOv5 algorithm trained with 4,530 immunofluorescence images to distinguish the degree of overlap between DAPI and c-Fos. We used Labelme software to get all the images in the dataset annotated by two experienced technicians, the dataset was divided into a training set (80%) and a test set (20%). We then used the test set to verify the performance of the model and achieved good results. Overall, our findings indicate that the YOLOv5 algorithm can quickly and efficiently identify the location and quantity of target proteins compared to the ImageJ method. These results suggest that the YOLOv5 algorithm, as a new method used in neuroscience, can save a considerable amount of time, provide feasible methods for quantitative statistics of proteins, and has good potential for application in the detection of target proteins in neuroscience.

This study also showed that the YOLOv5 algorithm, with its high speed, low background error detection rate, and strong versatility detection network, can detect and recognize target proteins. The recognition and quantitative statistics of proteins have significant implications in biology and neuroscience. The results of this study indicated that the YOLOv5 algorithm could accurately and quickly obtain the coordinates of the bounding box, confidence degree, and category probability of c-Fos images. These findings demonstrate the potential of the YOLOv5 algorithm as a detection approach for recognition and quantitative statistics of proteins.

Research on transfer learning shows that it can help small datasets achieve better results (40, 41). Therefore, we used a fine-tuned model in this task, which was trained from a public pre-trained model. It can handle similar identifying and quantifying tasks such as POMC proteins (Supplementary Figure 3). Biologists can simply input the original image into the model, and it will provide results that are automatically visualized in the output (Figure 2). People who want to use this tool need not understand any computer knowledge or change any parameters. The trained model can complete the work repeatedly and perfectly.

The common object in context (COCO) dataset is a public dataset obtained by the Microsoft team that can be used for image recognition, segmentation and captioning. This kind of data format is called COCO. COCO dataset proposes strict metrics for images of three different sizes (small, medium, and large): small targets (area < 32^2^), medium targets (32^2^ < area < 96^2^), and large targets (area > 96^2^). The area is measured as the number of pixels in the segmentation mask. In our datasets the size of nuclei with c-Fos was a small target. In state-of-the-art research on natural images, the performance of AP_small_ was always the worst. Only a few detectors (42–44) can exceed a value of 30 on this term, and AP_large_ can be approximately twice as much as AP_small_. This is because small targets have a low resolution, blurry images, and little information. Consequently, the feature expression ability is weak. In manual methods, c-Fos can be distinguished from an image by its special color and staining range. Further, it has no complicated background that influences fluorecytes. To solve this problem, we used the most direct method, enlarging the original images. This was done because the color and staining information are of great importance for detecting c-Fos. In data preprocessing, a trained SRGAN model was employed to rescale images, and the image clarity was dramatically increased compared to the normal resizing method. We also found that the preprocessing methods influenced the final metrics of the detection model (Figure 3).

Currently, there are several methods for the quantitative statistical analysis of proteins. Owing to the background of the images obtained and complexity of target proteins, manual counting, and ImageJ software are the two most commonly used methods for identifying and quantifying the number of target proteins. However, they have varying degrees of limitations in terms of processing speed and accuracy. We used manual counting, ImageJ, and YOLOv5 to process the same batch of images and the results showed that YOLOv5 took the least time and was more accurate (time: ImageJ software: 80.12 ± 1.67 s, manual counting: 3.41 ± 0.25 s, YOLOv5: 0.0251 ± 0.0003 s, *p* < 0.0001, *n* = 83; simple linear regression equation: ImageJ software: *Y* = 1.013 × *X* + 0.776, *R*^2^ = 0.837; manual counting: *Y* = 1.0*X + 0, *R*^2^ = 1; YOLOv5: *Y* = 0.9730*X + 0.3821, *R*^2^ = 0.933, *n* = 130, Figures 6B,C). These findings suggest that the application of YOLOv5 saves a significant amount time and improve the efficiency.

Various detection networks have been applied in different fields. They are mainly divided into two types: one-stage and two-stage detection. Among them, the YOLO (27) series and single-shot detector (SSD) (45) are typical representations of one-stage detection, and the faster region-based convolutional neural network (faster-RCNN) (46) is a typical representation of a two-stage detection model. Tahir et al. previously reported that the faster-RCNN had a higher accuracy (95.31%) than YOLO (94.2%) and SSD (84.61%) in satellite imagery to detect objects; however, YOLO is an obvious leader in terms of speed and efficiency (47). Alkentar et al. indicated that SSD had good detection ability but a high false positive ratio for drone detection. Moreover, the faster-RCNN had high recognition ability but long frame processing, and YOLO had high recognition rate and ability to work in real time (48). Therefore, two-stage detection has higher accuracy but slower speed, and one-stage detection achieves end-to-end training, which is faster but partially, sacrifices accuracy. After combining these characteristics of the identification network, we used YOLOv5 in this study, which has advantages in terms of speed and accuracy. Future improvements to the YOLO series or applications to other networks may result in faster and more accurate protein detection.

Owing to the limitation of image size and complexity of the processing steps, the processing speed of the ImageJ software is a disadvantage compared to the speed of manual counting. However, AI is superior to the other methods in terms of processing speed and accuracy. For YOLOv5, there are certain limitations. First, the collected dataset did not reflect all possible staining conditions. If the images contained a large area of the fluorescent band, our model could not classify c-Fos, and the same color band. Therefore, a test image was needed to ensure the quality of staining to avoid incorrect detection results that limited the range of applications. Second, the original images should be segmented for a convenient model learning. Third, a large number of protein annotations were required for training and prediction to improve identification accuracy, but the annotations may be biased because of the subjective evaluation of the two technicians. Finally, the three-layer or multilayer fluorescent staining images were not included in our study, which is important in neuroscience and biology. In future studies, we will require more technicians to annotate images to improve the accuracy of learning, collect a large number of multilayer fluorescent staining images to expand the range of applications, and improve the algorithm to increase the efficiency of recognition.

The YOLOv5 system can be applied to other proteins, and it could conceivably be extended to images of different types of brain tissue and peripheral tissue cells, not only optical images but also MRI images, positron emission tomography (PET) images and so on. In addition, the results can also apply to the field of neuroscience, e.g., DBS, optogenetic, TMS, ultrasound stimulation, and so on. Importantly, the YOLOv5 system could be applied to images of polychromatic immunofluorescence, which could detect and count multiple proteins simultaneously, as well as recognize various types of proteins, such as amyloid beta, tau, alpha-synuclein, and huntingtin. Our proposed method can thus aid the detection of proteins, which is of great significance for the early diagnosis, progression, assessment of curative effects, and prognostic evaluation of brain diseases, such as Alzheimer’s and Parkinson’s diseases. In conclusion, object detection algorithms have great potential in the field of neuroscience.

## Data availability statement

The raw data supporting the conclusions of this article will be made available by the authors, without undue reservation.

## Author contributions

YX and LX: conception and project administration. ZiL, KS, NP, ZhL, MP, XC, and XL: data curation. KS and NP: methodology and writing—original draft. All authors reviewed and commented on the manuscript.
