# Supplementary material for: Fast identification and quantification of c-Fos protein using you-only-look-once-v5
Source: Front Psychiatry. 2022 Sep 23;13:1011296. doi: 10.3389/fpsyt.2022.1011296 (PMC9537349; doi:10.3389/fpsyt.2022.1011296)
Supplement: Supplementary file 1 [file Data_Sheet_1.docx]

**Supplemental Information for:**

Fast Identification and Quantification of c-Fos protein using

YOLOv5

Na Pang^1,2^, Zihao Liu^3^, Zhengrong Lin^2^, Xiaoyan Chen^2^, Xiufang Liu^2^, Min Pan^3^, Keke Shi^2,*^, Yang Xiao^4,*^, Lisheng Xu^1,5,*^

^1^ The College of Medicine and Biological Information Engineering, Northeastern University, 195 Innovation Road, Shenyang, China, 110016

^2^ Institute of Biomedical and Health Engineering, Shenzhen Institutes of Advanced Technology, Chinese Academy of Sciences, 1068 Xueyuan Avenue, Shenzhen, China, 518055

^3^Shenzhen Hospital of Guangzhou University of Chinese Medicine (Futian)

^4^National Innovation Center for Advanced Medical Devices, Shenzhen, China

^5^Key Laboratory of Medical Image Computing, Ministry of Education, Shenyang, Liaoning 110169, China

**Supplementary Method:**

1. The specific processing steps of the Watershed algorithm available in ImageJ are as follows: 1) the image was imported into the ImageJ software, converted from RGB color into composite, and split into three channels; 2) the c-Fos and DAPI channels were translated to 8-bit images, respectively; 3) the threshold was adjusted and the background was removed to select targets by “Threshold”; 4) the image was converted into a binary image by applying threshold settings; 5) the holes were filled by “Process-Binary-Fill Holes”; 6) the overlap of the target was broke by “Process-Binary-Watershed”; 7) selections were created after the watershed setting, respectively; 8) selections were added in the region of interest (ROI) manager and the overlap was obtained by merged ROI; 9) overlapping selection was used to created mask, which was analyzed by combining the “Analyze Particles” function with a flexible preset size range and circularity to remove false positives and obtain the protein count.

2. POMC protein immunofluorescence images were collected from the Shenzhen Institutes of Advanced Technology, Chinese Academy of Sciences. The mice were sacrificed and brain tissues were obtained. The brain tissues were cut into slices of a 20-mm thickness and pre-rinsed three times with phosphate buffer saline. The slices were permeabilized and blocked at room temperature for 1 h and incubated with primary antibodies (POMC, Abcam, ab254257) at 4 °C for over 12 h. The slices were then washed thrice and incubated with secondary antibodies (488 donkey anti-rabbit, ThermoFisher, A21206) for 3 h. After washing, the slices were counterstained by 4′, 6-diamidino-2-phenylindole (DAPI). Images were acquired using a Nikon confocal microscope (ECLIPSE Ti2-U, Nikon, Japan).


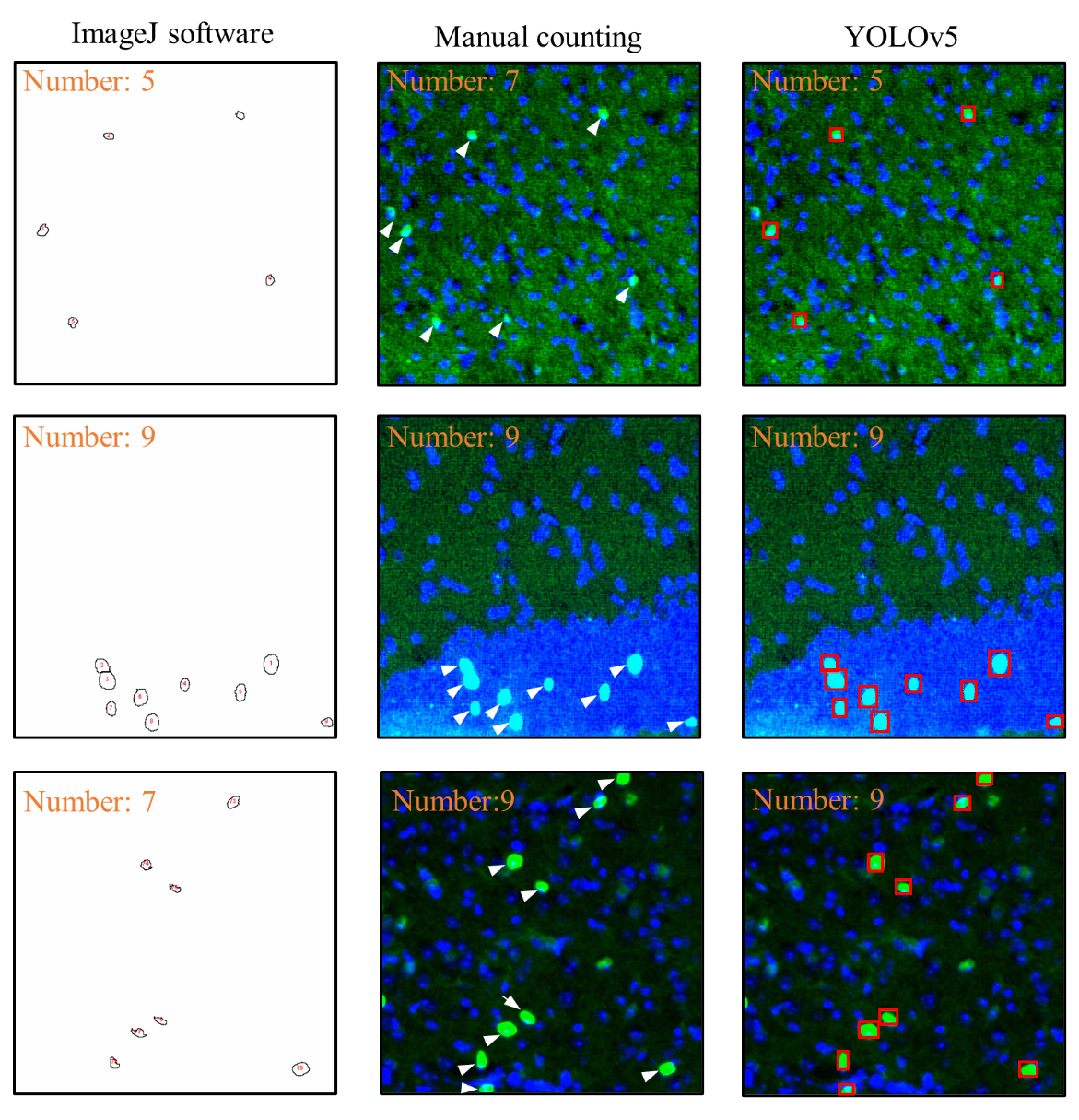


**Supplementary Figure 1.** Results of different recognition methods. Representative images of c-Fos recognition processed by ImageJ software (closed circles, Watershed algorithm), manual counting (white arrows) and YOLOv5 (red boxes).

**Supplementary Figure 2.** The number of c-Fos of Watershed algorithm and Threshold algorithm. The data shown represent the mean ± SD values for the indicated n (n = 10).


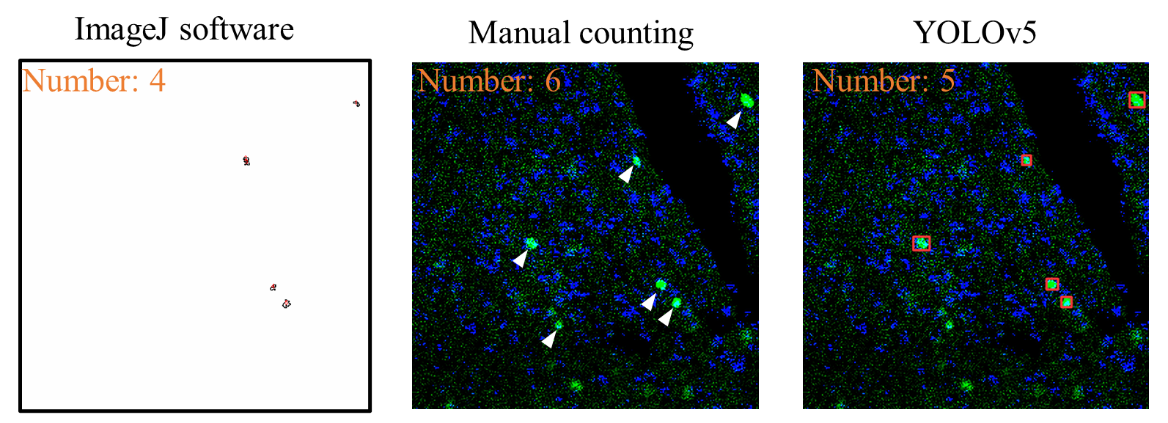


**Supplementary Figure 3.** Results of different recognition methods. Representative images of POMC recognition processed by ImageJ software (closed circles), manual counting (white arrows) and YOLOv5 (red boxes).
