# Supplementary figures and images for: Fast identification and quantification of c-Fos protein using you-only-look-once-v5
Source: Front Psychiatry. 2022 Sep 23;13:1011296. doi: 10.3389/fpsyt.2022.1011296 (PMC9537349; doi:10.3389/fpsyt.2022.1011296)

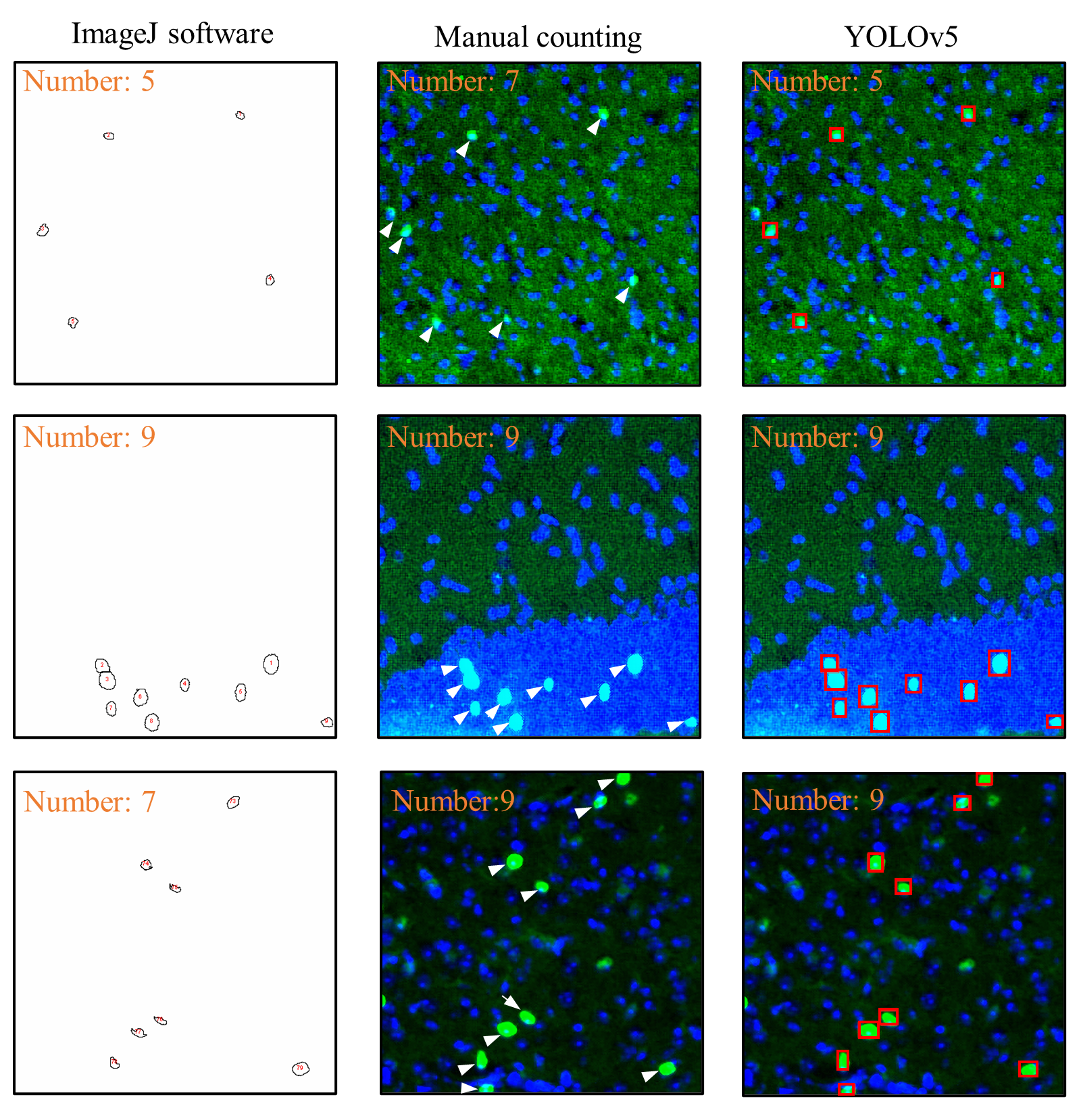

Supplement: Supplementary file 2 [file Image_1.TIF]

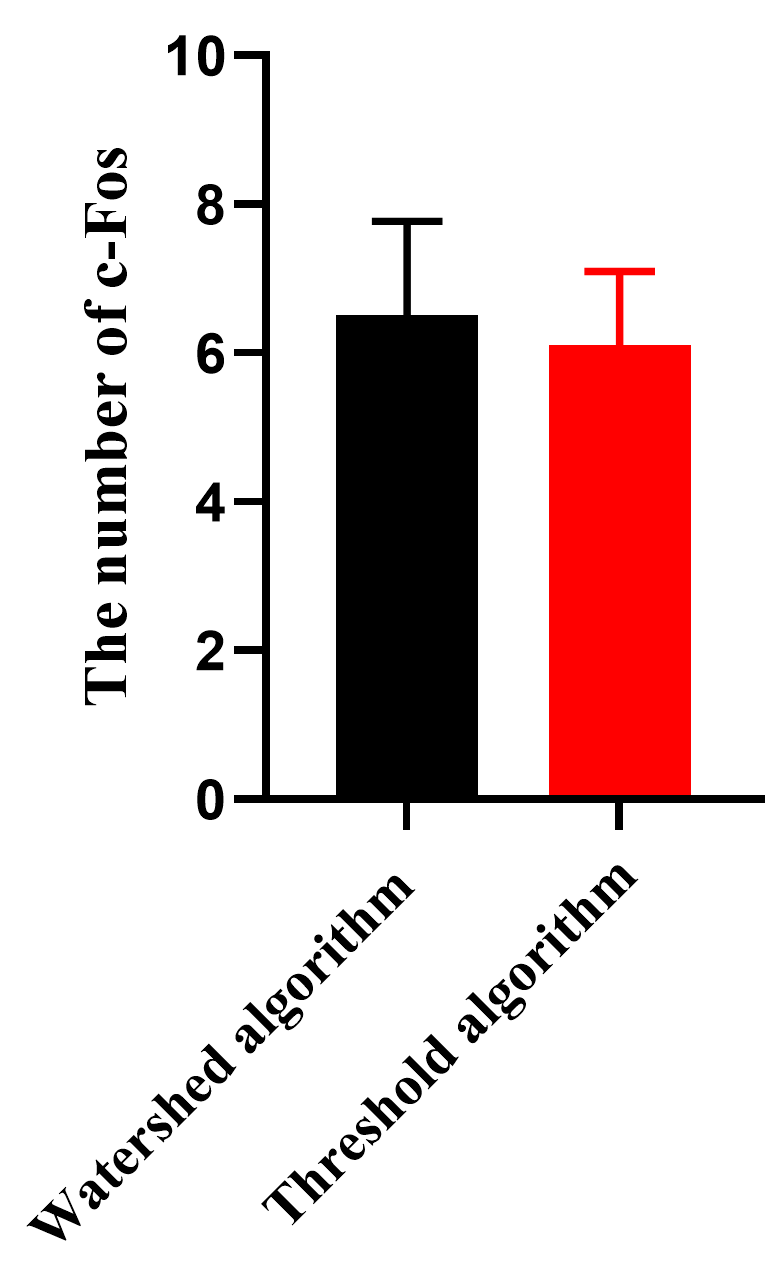

Supplement: Supplementary file 3 [file Image_2.TIF]

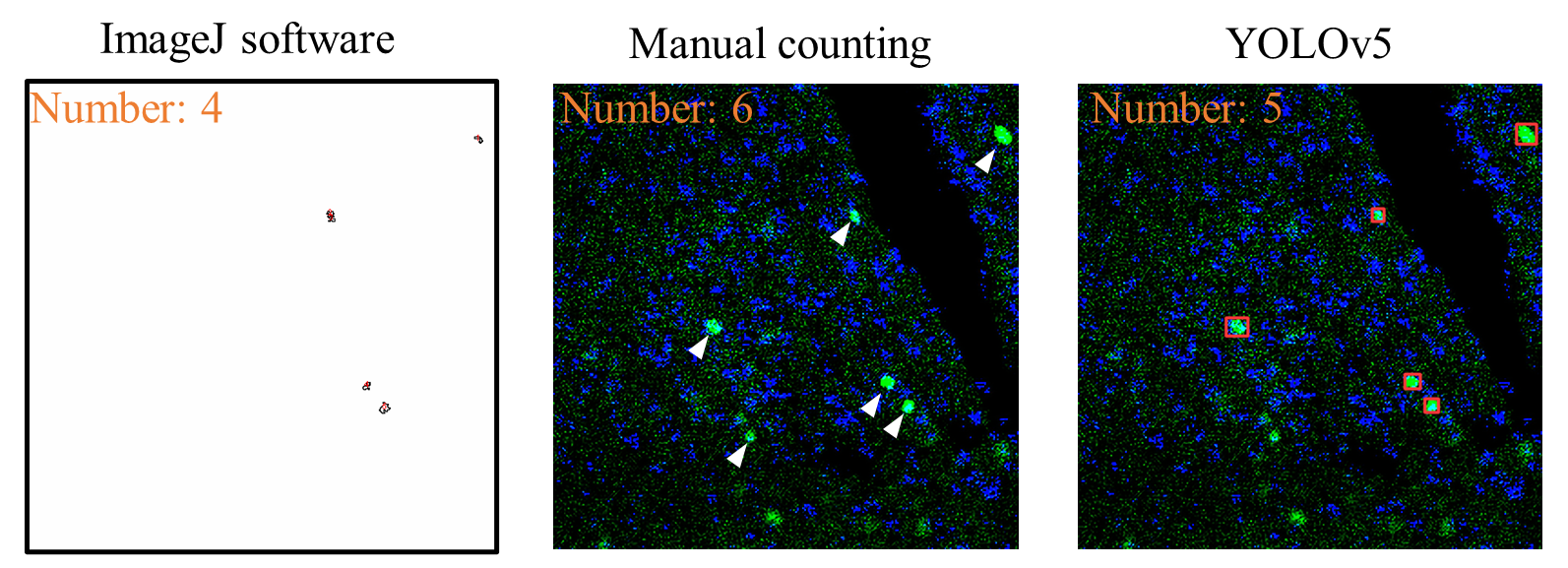

Supplement: Supplementary file 4 [file Image_3.TIF]
